# DGADiff: Decoupled Guide Attention with Diffusion Model for Portrait Stylization

**DOI:** 10.3390/s26092915

**Published:** 2026-05-06

**Authors:** Yi Ren, Zihan Shen, Junchao Fan, Guanlun Guo

**Affiliations:** 1 School of Transportation and Logistics Engineering, Wuhan University of Technology, Wuhan 430070, China; 2 School of Automotive Engineering, Wuhan University of Technology, Wuhan 430070, China; 3 School of Navigation Engineering, Wuhan University of Technology, Wuhan 430070, China

**Keywords:** decoupled guide attention, image generation, diffusion model, latent consistency model

## Abstract

Diffusion-based models have substantially propelled the progress of portrait stylization. Nevertheless, the lack of clear supervisory signals often leads to pattern drift in the target portrait. To overcome this issue, we introduce DGADiff, a training-free stylization framework based on a diffusion model. Specifically, we first leverage prior knowledge from a pre-trained latent consistency model (LCM) to efficiently sample representative features from noisy image pairs. Next, we design a Decoupled Guide Attention Mechanism (DGA), that disentangles the U-Net attention into separate self-attention and masked-attention tracks, enabling accurate transfer of fine-grained facial style patterns. Extensive experiments verify that our DGADiff achieves favorable results across multiple metrics in content-to-style and style-to-content multi-domain tasks, demonstrating the effectiveness of spatial attention decoupling for portrait stylization.

## 1. Introduction

Portrait transfer combines realistic facial features with styles from reference images, balancing identity preservation and style adaptation. While valuable for creative applications, achieving high-fidelity results remains challenging. This process not only preserves the essential features of the original portrait but also incorporates the unique stylistic patterns from the reference. It is a significant task in computer vision (CV) and image processing, with substantial impact on enhancing human recreational experiences and boosting the creative efficiency of artists. Despite significant progress in adjacent fields, such as image colorization [1,2], image retrieval [3,4], image translation [5], face anti-spoofing [6], cartoonization [7], and 3D portrait stylization [8], achieving high-fidelity, aesthetically pleasing portrait stylization remains a challenging goal. Traditional methods utilize filters [9], stochastic mathematical [10], and machine learning techniques [11], which makes it difficult to effectively distinguish and separate content and style in arbitrary images.

The advent of convolutional neural networks (CNNs) in deep learning brought new possibilities. Gatys et al. [12] first used a pre-trained visual geometry group network (VGGNet) [13] to accurately extract content and style features for style transfer, opening a precedent for image stylization. Since then, researchers have worked on VGGNet-based style transfer algorithms from various perspectives, progressively improving generation quality. The emergence of generative adversarial networks (GANs) [14] has further advanced the study of style transfer. Isola et al. [15] first used GAN to solve the mapping between arbitrary content domains and style domains, but it required paired training data. Zhu et al. [16] proposed a cyclic consistency generative adversarial network (CycleGAN) to achieve learning between arbitrary content-style domains without paired training data.

**Figure 1:**
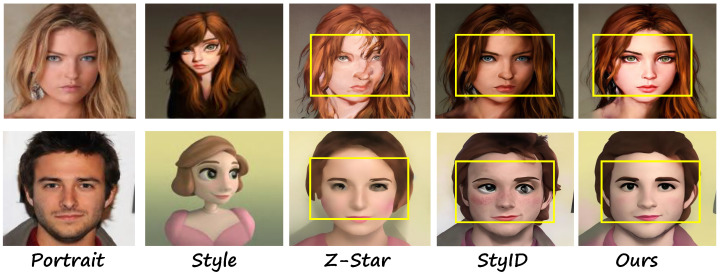
Generated images of Z-Star [18], StyID [19], and our method. The yellow boxes highlight regions where the previous methods suffer from facial pattern and color distribution inconsistency issues.

Inspired by the stable generation ability of the diffusion model (DM) [17], researchers have used it to achieve training-free image style transfer [18,19]. For example, StyID [19] used the pre-trained diffusion model to adjust the injection of the style features, achieving style information transfer while ensuring content retention. Z-star [18] developed a novel merge content-style attention control approach to transfer the complex style semantics. However, these methods apply style injection globally without explicit spatial decoupling—Z-Star merges content-style attention across all spatial tokens, while StyID modulates style injection intensity uniformly—causing foreground facial patterns to be contaminated by background style and leading to color distribution inconsistency in portrait results, as illustrated in Figure 1.

Consequently, to alleviate these issues, we propose a novel DGADiff, a training-free portrait stylization framework incorporating a Decoupled Guide Attention (DGA) Mechanism. It disentangles the U-Net attention into separate self-attention and masked-attention tracks, enabling accurate injection and transfer of fine-grained facial style characteristics without compromising global scene integrity. In addition, as an auxiliary processing step, we construct a Feature Orthogonal Fusion (FOF) strategy that combines frequency-domain wavelet decomposition with spatial mask-based orthogonal decomposition. Specifically, FOF applies discrete wavelet transform (DWT) to separate content structure (low-frequency) from style texture (high-frequency), while the DGA mechanism performs spatial foreground-background decoupling via SAM-generated masks. This two-level orthogonal design aims to reduce domain misalignment between content and style. Our contributions are:

- We propose a novel training-free diffusion model dubbed DGADiff for portrait style transfer to generate the stylized images with consistent style pattern;
- We design a masked Decoupled Guide Attention Mechanism (DGA) that decouples the self-attention and mask-attention tracks to improve the pattern consistency of stylized images;
- As an auxiliary design choice, we introduce a Feature Orthogonal Fusion (FOF) strategy that integrates frequency-domain wavelet decomposition with spatial mask-based orthogonal decomposition, aiming to reduce domain misalignment between content and style features;
- Extensive experiments verify that our DGADiff achieves favorable results on standard benchmarks, demonstrating the benefit of explicit spatial decoupling for portrait stylization.

## 2. Related Work

### 2.1. Image Style Transfer

Huang et al. [20] proposed the adaptive instance normalization (AdaIN) approach to synthesize images in real time by adjusting the first-order statistics between content and style features. However, it cannot take into account more complex higher-order statistics. Li et al. [21] proposed the whitening and coloring (WCT) method, which achieved fast style transformation by aligning the covariance statistics without training. However, it is prone to discordant stylistic textures. To avoid this issue, the SANet [22] uses the “global attention” approach [23] to dynamically transfer style information based on the content-style attention weight matrix. However, the local areas of the image generated by these methods are prone to the phenomenon of style pattern repetition. Recently, Zhu et al. [24] alleviated this pattern repetition issue by proposing a distributed and progressive attention mechanism (StyA2k), and AdaAttN [25] revisited the attention mechanism by jointly normalizing content and style features to better preserve local patterns. However, these methods are not effective in achieving satisfactory stylization of portrait images due to the inherent nature of portrait images.

### 2.2. Portrait Image Style Transfer

Portrait image style transfer, as a specialized subtask in image style transfer, focuses on transferring facial styles from reference images to portrait content while preserving content layout. In the field of CV, early works such as Pix2Pix [15], CycleGAN [16], and U-GAT-IT [26] were first proposed to achieve the information transfer of the target domain into the source domain. Subsequently, the large-scale StyleGAN [27] model was proposed, which modulates the synthesis of images by learning the latent features. Building upon the foundation of StyleGAN, AgileGAN [28], and DualStyleGAN [29] conducted fine-tuning operations to StyleGAN and introduced novel pathways to enhance facial consistency, but required a large dataset of training images. To mitigate the reliance on large target-domain datasets, StyleGAN-NADA [30] uses CLIP to guide zero-shot domain adaptation, and StyleGAN-Fusion [31] distills diffusion priors into StyleGAN for more flexible style adaptation. Score-distillation-based head stylization [32] further extends these ideas to 3D-aware portrait stylization. In contrast, JoJoGAN [33] devised a one-shot facial stylization approach. It generates a mixed-paired dataset of styles, thereby effectively enhancing the model’s performance in scenarios with limited samples. One-shot structure-aware stylized synthesis [34] also pursues a similar low-shot direction but requires additional per-style training.

### 2.3. Training-Free Diffusion-Based Portrait Stylization

Recently, several training-free methods have leveraged pre-trained diffusion models for portrait stylization. ZePo [35] adopts a multi-path latent consistency model framework with zero-shot sampling but applies standard self-attention without spatial decomposition, so foreground facial patterns can be contaminated by background style. StyID [19] modulates style injection intensity within a diffusion backbone, yet its modulation is spatially uniform and lacks explicit region awareness. Z-Star [18] proposes a merged content-style attention control strategy operating globally across all tokens, without distinguishing between face and background regions. Portrait Diffusion [36] employs a chain-of-painting approach with DDIM inversion under a standard SD 1.5 backbone, which differs from LCM-based frameworks in both scheduler and step count. Beyond portrait-specific work, inversion-based style transfer with diffusion [37] and general image-to-image translation with one-shot image guidance [38] employ diffusion inversion and guidance to inject style information; attention-sharing techniques such as MasaCtrl [39] and StyleAligned [40] modify self-attention for consistent multi-image synthesis but are not tailored to foreground–background decoupling for portrait stylization.

A common limitation of these methods is the absence of **explicit spatial decoupling**: they treat all spatial tokens uniformly, which leads to foreground–background style interference in portrait images. In contrast, our DGA mechanism introduces three architectural distinctions: (1) a **dual-branch attention design** that separates self-attention (preserving background coherence) from Masked Guide-Attention (targeting foreground style transfer); (2) **SAM-mask-based spatial gating** that explicitly isolates foreground and background regions; and (3) **AdaIN alignment on Q/K** to bridge the statistical gap between content and style domains before attention computation. These structural differences position DGA as a spatially aware attention mechanism specifically designed for portrait stylization, rather than an incremental improvement over existing global-attention approaches.

## 3. Our Methods

Building on denoising diffusion probabilistic models (DDPM) [41] and their deterministic accelerated variant DDIM [42], we leverage prior knowledge from a pre-trained Latent Consistency Model (LCM) [43] to build DGADiff, a training-free, multi-branch framework for portrait stylization. During encoding, the content image $I_{c}$ and style image $I_{s}$ are processed by the VAE encoder *E* to obtain content features $f_{c}$ and style features $f_{s}$. The generation stage employs three distinct paths: C2C and S2S reconstruct their respective domains through forward–reverse diffusion. C2S (Content-to-Style) injects features from the other two paths into a Decoupled Guide Attention Mechanism (DGA) module for style transfer. Finally, the stylized features are decoded to generate image $I_{cs}$.

### 3.1. Framework Overview

**Figure 2:**
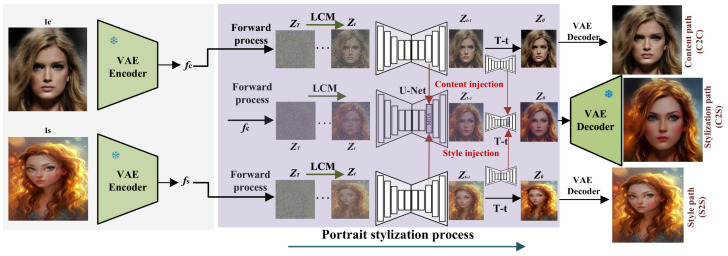
**High-level overview of the DGADiff framework.** The content image $I_{c}$ and style image $I_{s}$ are encoded by a frozen VAE encoder into latent features $f_{c}$ and $f_{s}$. Three parallel LCM-based diffusion paths—Content (C2C), Stylization (C2S), and Style (S2S)—process the latents simultaneously. The C2S path receives content injection from C2C and style injection from S2S through the Decoupled Guide Attention (DGA) module (detailed in Figure 3); an ❄ marks the timesteps at which DGA injection is applied. The stylized output is decoded by the VAE decoder.

In this work, our main insight is to mine the prior knowledge from the pre-trained latent consistency model (LCM) [43] to develop a novel zero-shot multi-paths model for portrait stylization, named DGADiff. Figure 2 shows the overview framework of our proposed DGADiff.

**Figure 3:**
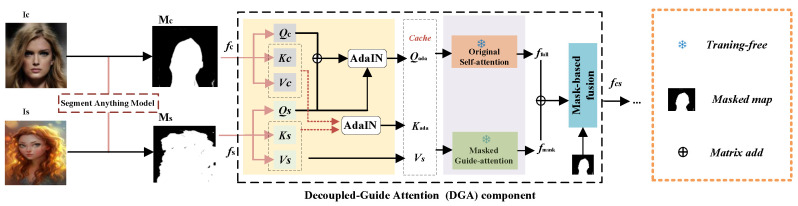
**Detailed architecture of the Decoupled Guide Attention (DGA) component.** Content and style features ($f_{c}$, $f_{s}$) are projected into Q/K/V triplets. AdaIN aligns Q and K across domains to produce $Q_{\mathrm{ada}}$ and $K_{\mathrm{ada}}$. Two parallel branches—original self-attention (producing $f_{\mathrm{full}}$) and Masked Guide-Attention (producing $f_{\mathrm{mask}}$ using SAM-generated masks $M_{c}$, $M_{s}$)—are combined via mask-based fusion to yield the final fused feature $f_{cs}$, which is fed into the subsequent denoising U-Net layers. In the diagram, solid colored arrows denote the main feature flow (content path in blue, style path in orange, fused path in red), while dashed arrows denote mask alignment and broadcasting.

Specifically, our proposed DGADiff consists of the VAE encoder *E*, diffusion generation process, and VAE decoder *D*. In the encoding process, given the content image $I_{c}$ and style image $I_{s}$, we can feed them into the encoder to extract the content feature $f_{c}$ and style feature $f_{s}$. In the diffusion generation process, we build three main diffusion paths: the content path (C2C), the stylization path (C2S), and the style path (S2S). The content path takes the data distribution of the potential content feature as $z_{0}$ in the diffusion mechanism and reconstructs the content image after forward and reverse diffusion. The same is true for the style path. For the stylization path, the content and style paths provide the injected content-style features for the Decoupled Guide Attention (DGA) component designed (as shown in Figure 3). In the decoding process, the obtained stylized feature by the diffusion generation process is fed into the VAE decoder *D* to output the stylized image $I_{cs}$. In the following, we describe the core contributions, the Decoupled Guide Attention (DGA) component.

### 3.2. Decoupled Guide Attention Mechanism (DGA)

**Definition of Training-free Boundary:** Before delving into the details, we explicitly define the boundary of our “training-free” claim. Throughout the entire inference process of DGADiff, no network parameters—including the VAE, Diffusion U-Net, LCM scheduler, and the auxiliary Segment Anything Model (SAM)—are updated. The stylization is achieved purely through forward inference, latent inversion, and intermediate feature recombination without any extra fine-tuning.

Existing methodologies [18,19] have shown that style transfer can be achieved by integrating style information from the style path and content semantics from the content path into specified attention layers of the stylization path’s U-Net. The standard cross-attention mechanism can be formally written as:

(1) $$\mathrm{Attn}\left(Q,K,V\right)=\mathrm{Softmax}\left(\frac{QK^{⊤}}{\sqrt{d}}\right)V,$$

where $Q,K,V\in R^{N\times d}$ denote the Query, Key, and Value matrices, respectively. N=h×w is the sequence length of the flattened spatial tokens, and *d* is the dimension of the attention head.

However, portrait images often exhibit abundant and complex style patterns specifically in the foreground areas. A naive application of the original attention approach struggles to accurately map style into specific content layouts. To overcome this, we design a Decoupled Guide Attention Mechanism (DGA), the structure of which is illustrated in Figure 3.

Specifically, at a given U-Net layer, we first extract the original Query, Key, and Value features from the content path ($Q_{c},K_{c},V_{c}$) and the style path ($Q_{s},K_{s},V_{s}$). To balance the fusion of content and style features, we perform a matrix summation of $Q_{c}$ and $Q_{s}$, and apply the affine transformation of AdaIN [20] to align the statistical distribution, yielding a new query matrix $Q_{ada}$. An identical AdaIN operation is applied to the Key matrices:

(2) $$\begin{array}{ccc} \bar{Q} & =Q_{c}+Q_{s},\\ Q_{ada} & =\mathrm{AdaIN}\left(\bar{Q},Q_{s}\right),\;K_{ada} & =\mathrm{AdaIN}(K_{c},K_{s}). \end{array}$$

**Justification for AdaIN on Q and K only:** In the attention mechanism, Q and K jointly determine the attention map (i.e., *where* to attend), while V carries the feature content (i.e., *what* to retrieve). Applying AdaIN on Q and K aligns the statistical distributions of content and style features in the attention similarity space, so that the attention map reflects semantic similarity rather than domain-specific statistical biases. The V matrix carries the actual style information ($V_{s}$); applying AdaIN on V would distort the style features themselves, defeating the purpose of style transfer.

**Mask Alignment and Decoupling Strategy:** To provide precise spatial supervision, we decouple the attention computation into two parallel branches: an original self-attention branch and a masked-guide attention branch. We employ SAM to generate binary spatial masks for the content and style images, denoted as $M_{c},M_{s}\in {\left\{0,1\right\}}^{H\times W}$. Since the attention operates on downsampled token sequences, we apply nearest-neighbor interpolation to resize the masks to h×w and flatten them into 1D vectors $M_{c},M_{s}\in {\left\{0,1\right\}}^{N\times 1}$ to geometrically align with the features.

For the masked-guide branch, the features are masked using element-wise multiplication (⊙) with broadcasting along the channel dimension, scaled by a weighting factor λ to emphasize foreground patterns. Meanwhile, the unmasked versions are retained for the original self-attention branch to preserve background structural consistency:

(3) $$\begin{array}{cccc} Q_{m} & =\lambda (Q_{ada}⊙M_{c}),\; & K_{m} & =\lambda (K_{ada}⊙M_{s}),\\ Q_{f} & =Q_{ada},\; & K_{f} & =K_{ada}. \end{array}$$

Finally, the style facial information stored in $V_{s}$ is injected into both branches via attention computation. A naive combination would linearly weight the outputs of these two branches with a scalar hyperparameter. However, this ignores semantic correlations within the content layout. Therefore, we utilize the aligned content mask $M_{c}$ as a spatial gating matrix to smoothly blend the attention outputs. The final output feature $f_{cs}\in R^{N\times d}$ of our DGA module is defined as:

(4) $$\begin{array}{cc} f_{cs}= & M_{c}⊙\mathrm{Attn}\left(Q_{m},K_{m},V_{s}\right)\\ \; & +\left(1-M_{c}\right)⊙\mathrm{Attn}\left(Q_{f},K_{f},V_{s}\right). \end{array}$$

**Theoretical Justification for Mask Decoupling.** The mask-based decoupling in Equation (4) provides two design properties: (1) *Foreground isolation*—the masked branch operates on mask-gated features so that primarily foreground-relevant tokens contribute to the attention map, concentrating the attention on facial regions; (2) *Background preservation*—the unmasked branch retains full spatial coverage and its output is gated by $\left(1-M_{c}\right)$ to affect only background regions, helping preserve structural coherence. Intuitively, mask gating zeroes out irrelevant key–query interactions, reducing the effective receptive field. We empirically observe that the attention entropy of the masked branch is lower than the unmasked branch, translating to more precise style pattern assignment in facial areas.

**SAM Integration.** We employ the Segment Anything Model (SAM) [44] in a zero-shot manner with automatic point-grid prompting. Given an input image of 512×512 resolution, SAM generates candidate masks; we select the mask with the highest predicted IoU score covering the facial region. The resulting binary mask is downsampled via nearest-neighbor interpolation to match each U-Net layer’s spatial resolution. SAM’s parameters remain completely frozen throughout inference.

**Injection Layer Selection.** We inject DGA at the Mid and Late (Up) blocks of the U-Net. Early (Down) blocks encode high-resolution, content-specific features that lack sufficient semantic abstraction for cross-domain style mapping. The Mid block captures global semantic context at the coarsest resolution, enabling robust content-style correspondence. The Late blocks progressively render fine-grained style details. As confirmed in our ablation study (Section 4.4), Mid + Late injection achieves a favorable trade-off between quality and speed compared to Early-only or All-layer injection.

To clearly demonstrate the overall inference flow, including the three parallel paths (C2C, S2S, C2S) and the specific injection sequence of our DGA module, we summarize the stylization procedure in Algorithm 1.
**Algorithm 1** Training-free Portrait Stylization via DGADiff**Require:** 
Content image $I_{c}$, Style image $I_{s}$, Pre-trained VAE, SAM, LCM U-Net $\epsilon_{\theta }$, DGA injection layers *L*, LCM steps *T*, Mask weight λ.**Ensure:** 
Stylized portrait image $I_{cs}$.  1:**Initialize:** Freeze all model parameters.  2:*// SAM preprocessing: binary masks $M_{c},M_{s}\in {\left\{0,1\right\}}^{H\times W}$*  3:Extract spatial masks: $M_{c}\leftarrow \mathrm{SAM}\left(I_{c}\right)$, $M_{s}\leftarrow \mathrm{SAM}\left(I_{s}\right)$.  4:*// VAE encoding: latents $z_{T}^{c},z_{T}^{s}\in R^{1\times 4\times h\times w}$*  5:Encode images to latents: $z_{T}^{c}\leftarrow {\mathrm{VAE}}_{enc}\left(I_{c}\right)$, $z_{T}^{s}\leftarrow {\mathrm{VAE}}_{enc}\left(I_{s}\right)$.  6:Initialize stylization latent $z_{T}^{cs}\in R^{1\times 4\times h\times w}$.  7:**for** each timestep t=T,T−1,…,1 **do**  8:   *// C2C path: content reconstruction (fixed timestep)*  9:   $\left\{Q_{c},K_{c},V_{c}\right\}\leftarrow \epsilon_{\theta }\left(z_{t}^{c},t\right)$    *// each $\in R^{N\times d}$, $N\;=\;h_{l}w_{l}$*10:   *// S2S path: style reconstruction (fixed timestep)*11:   $\left\{Q_{s},K_{s},V_{s}\right\}\leftarrow \epsilon_{\theta }\left(z_{t}^{s},t\right)$    *// each $\in R^{N\times d}$*12:   *// C2S path: stylization (actual denoising schedule)*13:   **for** each layer *l* in U-Net **do**14:     **if** l∈L (Target Injection Layers) **then**15:        Downsample & flatten $M_{c},M_{s}\to M_{c},M_{s}\in {\left\{0,1\right\}}^{N\times 1}$.16:        Compute $Q_{ada},K_{ada}$$\in R^{N\times d}$ via Equation (2).17:        Masked branch: $Q_{m}\;=\;\lambda \left(Q_{ada}⊙M_{c}\right)$, $K_{m}\;=\;\lambda \left(K_{ada}⊙M_{s}\right)\in R^{N\times d}$.18:        Unmasked branch: $Q_{f}\;=\;Q_{ada}$, $K_{f}\;=\;K_{ada}\in R^{N\times d}$.19:        Compute $f_{cs}$$\in R^{N\times d}$ via Equation (4); replace attention output.20:     **else**21:        Perform standard self/cross-attention.22:     **end if**23:   **end for**24:   Update latents $z_{t-1}^{c},z_{t-1}^{s},z_{t-1}^{cs}$$\in R^{1\times 4\times h\times w}$ via LCM scheduler.25:**end for**26:Decode: $I_{cs}\leftarrow {\mathrm{VAE}}_{dec}\left(z_{0}^{cs}\right)$.27:**return** $I_{cs}$

### 3.3. Feature Orthogonal Fusion (FOF)—Auxiliary Processing

As an auxiliary processing step to reduce domain misalignment between content and style features, we introduce the Feature Orthogonal Fusion (FOF) strategy. Note that our ablation study (Section 4.4) demonstrates that the primary performance driver of DGADiff is the DGA mechanism; the wavelet frequency-domain component of FOF provides a limited additional benefit. FOF operates on two orthogonal dimensions: frequency-domain decomposition and spatial mask-based decomposition.

**Frequency-Domain Orthogonal Processing.** We apply discrete wavelet transform (DWT) to decompose the content latent $z^{c}$ and style latent $z^{s}$ into frequency sub-bands:

(5) $$\left(z_{L}^{c},z_{H}^{c}\right)=\mathrm{DWT}\left(z^{c}\right),\;\left(z_{L}^{s},z_{H}^{s}\right)=\mathrm{DWT}\left(z^{s}\right),$$

where $z_{L}$ and $z_{H}$ denote the low-frequency (approximation) and high-frequency (detail) sub-bands, respectively. We suppress high-frequency components in the content latent (preserving structural layout) and suppress low-frequency components in the style latent (preserving style textures):

(6) $${\hat{z}}^{c}=\mathrm{IDWT}\left(z_{L}^{c},\alpha \cdot z_{H}^{c}\right),\;{\hat{z}}^{s}=\mathrm{IDWT}\left(\beta \cdot z_{L}^{s},z_{H}^{s}\right),$$

where α<1 attenuates content high-frequency noise and β<1 attenuates style low-frequency structure. This is designed so that content contributes primarily structural information (low-frequency) while style contributes primarily textural patterns (high-frequency), promoting frequency-domain orthogonality.

**Spatial Orthogonal Decomposition.** Complementing the frequency-domain processing, the DGA mechanism (Section 3) performs spatial orthogonal decomposition using SAM-generated binary masks $M_{c}$ and $M_{s}$. The foreground (face) and background regions are processed through separate attention branches, as formalized in Equation (4).

**Overall FOF Integration.** The frequency-processed latents ${\hat{z}}^{c}$ and ${\hat{z}}^{s}$ are used as inputs to the three-path diffusion process, where the DGA module further performs spatial orthogonal fusion. The combined FOF strategy aims to keep content structural information and style pattern information “orthogonal” (non-interfering) throughout the generation pipeline.

## 4. Experiments

### 4.1. Experimental Setup

DGADiff is implemented in PyTorch 2.1.0 (CUDA 12.1) and evaluated on a single NVIDIA RTX 4090 GPU (24 GB). We adopt the latent consistency model (LCM) [43] as the backbone with 4 sampling steps, a null text prompt, and classifier-free guidance [45] with a scale of 2. For evaluation, we use portrait-style images from AAHQ [46] and portrait-content images from CelebA-HQ [47], all resized to 512×512. No parameter updates are performed at any stage; the entire pipeline is training-free.

#### Evaluation Metrics

We adopt four complementary metrics. **FID** [48] and **ArtFID** [49] (↓) measure the distributional distance between generated and reference images, capturing overall visual quality; these Fréchet-distance metrics are closely related to MMD-based distributional criteria analyzed by Bińkowski et al. [50]. **FaceSim** [51] (↑) computes the cosine similarity of ArcFace identity embeddings between the generated image and the content input, quantifying identity preservation. **CLIP-I** [52] (↑) measures the CLIP-space cosine similarity between the generated image and the style reference, reflecting style consistency. These four metrics are complementary to classical low-level image-quality indices such as the universal image quality index [53] and SSIM [54], as well as deep perceptual metrics such as LPIPS [55]. Together, they evaluate content retention, style transfer fidelity, and perceptual quality.

### 4.2. Qualitative Comparison

**Figure 4:**
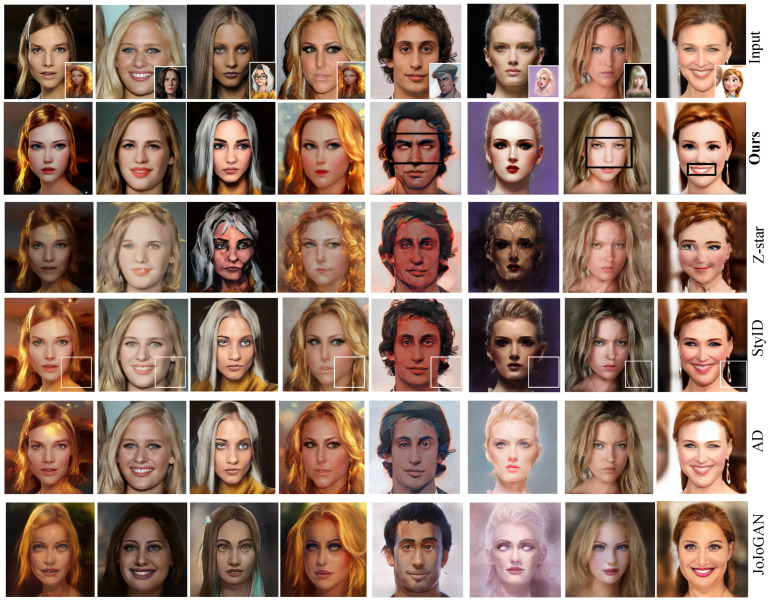
**Comparison with previous transfer methods.** In each row, the first column shows the content image, the second column shows the style reference, and the remaining columns show the stylized results of our method and four baselines. The boxes highlight representative regions for visual comparison, including facial pattern consistency, identity preservation, and background style leakage.

We compare DGADiff with four representative methods: JoJoGAN [33], Z-Star [18], StyID [19], and AD [56]. Figure 4 shows representative results.

Z-Star (Figure 4, 3rd column) manipulates content-style attention weights directly but lacks a portrait-specific strategy, leading to poor facial identity preservation—strong style transfer comes at the cost of structural fidelity. StyID (4th column) retains content semantics but fails to capture fine-grained facial style patterns from the reference, limiting its effectiveness for portrait stylization. AD (5th column) leverages attention distillation losses and produces visually clean outputs, yet it does not reproduce the style patterns of the reference portrait. JoJoGAN (6th column) fine-tunes StyleGAN [27] on the target style and achieves recognizable style similarity, but requires per-style optimization.

In contrast, DGADiff (Figure 4, 2nd column) renders rich facial style patterns while preserving the content layout and overall visual fidelity. Notably, even compared with the recent AD [56], DGADiff shows stronger preservation of fine-grained portrait patterns in these examples without requiring any training.

### 4.3. Quantitative Evaluation

To rigorously assess the performance of our proposed DGADiff and address the potential metric mismatch in style transfer tasks, we strictly align our evaluation protocol with the task objectives. Specifically, we categorize our quantitative metrics into three distinct dimensions: (1) **ID Preservation**: We use FaceSim [51] (↑) to measure the identity feature cosine similarity strictly between the *generated image* and the *content input*. (2) **Style Consistency**: We introduce CLIP-I [52] (↑) to evaluate the semantic style alignment strictly between the *generated image* and the *style input*. (3) **Perceptual Quality and Distribution**: We employ ArtFID [49] (↓) and FID [48] (↓) to measure the overall generation quality.

**Experimental Setup:** To ensure comprehensive evaluation, we conduct experiments under two different settings. *Setting 1 (Standard 100-image benchmark):* We randomly select 10 content portrait images from CelebA-HQ and 10 style images from AAHQ, generating a total of 100 stylized images per method. This setting primarily focuses on strict pairwise content-style alignment (FaceSim, CLIP-I). *Setting 2 (Large-scale 1000-image benchmark):* It is well known that distribution-based metrics like FID and ArtFID are statistically unstable and exhibit high variance when computed on small sample sizes (e.g., 100 images). To ensure the statistical reliability of these specific metrics, we design a large-scale benchmark by selecting 50 content images and 20 style images (generating 1000 images per method). To further eliminate randomness, the FID and ArtFID scores in this setting are calculated over three independent random sampling runs, reporting the *mean ± standard deviation*.

**Results Analysis.** Table 1 and Table 2 summarize the quantitative results. Across both settings, DGADiff acquires the highest FaceSim and CLIP-I scores, obtaining higher values than both optimization-based (JoJoGAN) and training-free (StyID) baselines on these benchmarks. This indicates that the DGA mechanism helps preserve content identity while transferring style patterns via AdaIN-aligned attention.

To mitigate the statistical instability of distribution metrics on small sample sizes, Table 2 reports results on the 1000-image benchmark with mean ± std over three runs. DGADiff achieves the lowest ArtFID (24.95±0.35) and FID (15.24±0.28) with smaller variance than the baselines, suggesting that it produces more consistent output quality across diverse content-style combinations.

**Figure 5:**
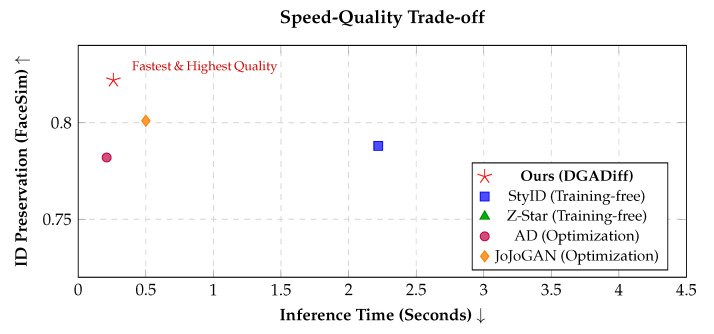
The speed–quality trade-off curve comparing our DGADiff with existing state-of-the-art methods. Each marker corresponds to one method (Z-Star: green triangle; StyID: blue square; AD: purple circle; JoJoGAN: orange diamond; ours: red star). Our method (red star) is situated in the favorable top-left region (faster inference, higher FaceSim), demonstrating competitive ID preservation at a near-real-time inference speed (0.26 s) without any parameter training.

**Efficiency and Fairness Analysis.** The inference times reported in Table 1 and Table 2 and Figure 5 measure *diffusion-only* time (excluding SAM preprocessing and VAE encode/decode) on an NVIDIA RTX 4090 at 512×512 resolution, following the convention of the compared methods. Under this scope, DGADiff runs in 0.26 s per image. We additionally profile the full end-to-end pipeline on an RTX 5090 (including VAE and SAM), which completes in ∼2.3 s per image. Because the two measurements differ in both GPU and timing scope, they should not be compared directly.

For the cross-method comparison in Table 1 and Table 2, all baselines use their published timing figures obtained on RTX 4090 hardware. Our DGADiff timing is measured under the same GPU and resolution. Diffusion-based baselines share the same LCM backbone with 4 inference steps where applicable.

To visualize the trade-off, we plot the speed–quality curve in Figure 5. DGADiff (red star) occupies the top-left region, combining high ID preservation with fast inference. Compared to Z-Star (3.85 s) and StyID (2.22 s), DGADiff is roughly 9× and 8.5× faster, respectively, owing to its streamlined parallel-path design and direct DGA injection without iterative latent optimization. The optimization-based AD [56] is marginally faster (0.21 s) but shows lower FaceSim (0.782 vs. 0.822) and requires per-style training. Overall, Figure 5 shows that DGADiff achieves competitive stylization quality at practical inference speed.

### 4.4. Ablation Experiment

To comprehensively validate the effectiveness and rationality of our DGADiff, we conduct extensive ablation studies. These studies not only verify the necessity of core modules but also explore key architectural designs, including mask fusion strategies, injection layer locations, and hyperparameter sensitivity. All ablations are evaluated under the standard 100-image setting. The quantitative results are summarized in Table 3.

**Figure 6:**
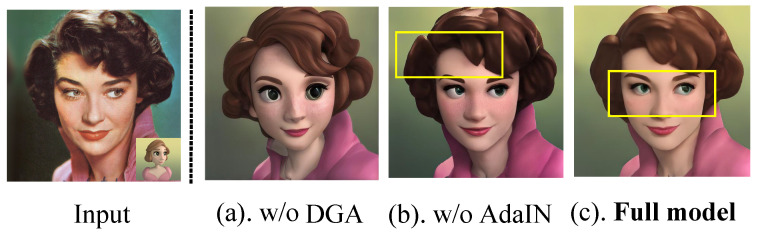
Ablation experiments of DGADiff. The two panels visualize the effects of removing key components of our method, with yellow boxes highlighting the affected facial regions. (**a**) Removing the full DGA module causes facial style bleeding into the background. (**b**) Removing the AdaIN alignment leads to inaccurate color rendering in the facial region. (**c**) Full model.

**(1) Effectiveness of Core Components:** We first remove the entire DGA module (replacing it with standard cross-attention). This ablation yields a ZePo-like [35] controlled configuration under our unified backbone and evaluation setting. As shown in Table 3a, the FaceSim drops to 0.797, and ArtFID increases to 28.54. Visually (see Figure 6a), this leads to a noticeable shift in facial style patterns and style bleeding into the background. This confirms that DGA effectively restricts style rendering to specific facial areas. Next, we remove the AdaIN feature alignment. The rendering of style colors becomes inaccurate (Figure 6b), and the FID metric degrades (19.67), proving that AdaIN is crucial for bridging the statistical gap between content and style domains.

**(2) Mask Fusion Strategies:** In our DGA, we utilize the content mask $M_{c}$ for spatial gating (Equation (4)). To validate this design choice, we compare it against two alternatives in Table 3b: *Linear Weighting* (using a global scalar α=0.5 without spatial masks) and *Concatenation* (concat along channel dim followed by a linear layer). Spatial Gating achieves the best balance. Linear weighting causes global style ghosting (worse ArtFID), while concatenation destroys the spatial layout (FaceSim drops to 0.790).

**(3) Impact of Injection Layers:** The U-Net consists of Down (Early), Mid, and Up (Late) blocks. Where we inject the DGA module, it significantly affects both quality and inference efficiency. As shown in Table 3c, injecting only in *Early* layers maintains high ID preservation but fails to transfer fine style textures (ArtFID 29.85). Injecting in *All* layers yields marginal quality improvements over *Mid + Late* but increases the inference time and VRAM usage. To achieve a favorable speed-quality trade-off, DGADiff injects DGA into the *Mid and Late (Up)* blocks, securing competitive generation quality while keeping inference fast (0.26 s).

**(4) Sensitivity of Gating Weight (λ):** The parameter λ controls the intensity of the masked style features. Table 3d reports the sensitivity analysis. A smaller λ (e.g., 0.5) leads to under-stylization (highest FaceSim but worst ArtFID). Conversely, an overly large λ (e.g., 1.5) causes structural artifacts and identity loss (FaceSim drops to 0.775). Setting λ=1.0 yields the most harmonious stylization results.

### 4.5. Discussion: Generalizability of DGA

The DGA module operates at the self-attention layer level within the U-Net, which is a standard architectural component shared across virtually all latent diffusion models. This makes DGA inherently architecture-agnostic: it directly applies to any Stable Diffusion variant (SD 1.5/2.1/XL), is compatible with any noise scheduler (DDPM, DDIM, DPM-Solver, LCM), and can be combined with ControlNet without conflict since ControlNet provides structural conditioning via residual connections while DGA modifies self-attention maps. Extending DGA to newer architectures such as DiT (Diffusion Transformer) would require adapting the mask injection to transformer attention blocks, which we consider as future work.

### 4.6. Limitations

While DGADiff achieves strong performance, several limitations remain. First, our method relies on SAM-generated masks for spatial decoupling; when SAM fails to produce accurate face segmentation (e.g., due to extreme occlusions, unusual poses, or artistic content images), the mask quality degrades and DGA’s spatial gating becomes less effective. Second, the current implementation assumes a single primary face per image; multiple faces may receive inconsistent stylization. Third, extreme side profiles (>$60^{\circ }$) and heavy occlusions reduce the effective face mask area, limiting fine-grained style transfer in peripheral facial regions. Fourth, highly abstract or non-representational art styles (e.g., cubism) challenge the content-style attention correspondence, as the semantic gap exceeds the capacity of AdaIN alignment alone. Addressing these failure modes is left to future work.

### 4.7. Ethical Considerations

This work processes real human facial images from CelebA-HQ and AAHQ, both publicly available research datasets with established terms of use. We acknowledge the dual-use risks inherent in face generation technology: portrait stylization could potentially be misused for non-consensual likeness manipulation or deepfake creation. We strongly oppose such misuse and advocate for responsible deployment with appropriate safeguards, including user consent verification, watermarking of generated images, and compliance with local regulations on synthetic media. DGADiff is designed for creative and artistic applications, not identity impersonation or deceptive content generation.

## 5. Conclusions

In this work, we propose a novel training-free DGADiff framework, which effectively addresses the issues of pattern shift and color inconsistency in portrait stylization. Through the design of the Decoupled Guide Attention (DGA) mechanism, precise facial style detail transfer and complete content preservation are achieved. Additionally, the auxiliary Feature Orthogonal Fusion (FOF) strategy combines frequency-domain wavelet decomposition with spatial mask-based orthogonal decomposition, aiming to reduce domain misalignment between content and style. We leverage the prior knowledge of the pre-trained latent consistency model (LCM) for efficient feature sampling. Extensive experiments demonstrate that DGADiff achieves favorable results compared with existing methods in terms of identity preservation, style consistency, and perceptual quality, showing consistent performance and high-quality style restoration.

## Figures and Tables

**Table 1:** Quantitative comparison with previous methods on the standard benchmark (**100 generated images**). **Bold** and underline indicate the best and second-best results, respectively. Note: Distribution metrics (ArtFID, FID) here are computed on a small sample size for preliminary reference.

Methods	ID Preservation	Style Consistency	Perceptual Distribution		Inference Time (s) ↓	Training-Free?
	FaceSim ↑	CLIP-I ↑	ArtFID ↓	FID ↓		
JoJoGAN [33]	0.806	0.645	31.42	20.11	0.50	×
Z-Star [18]	0.739	0.680	29.15	19.11	3.85	✓
StyID [19]	0.794	0.715	28.02	19.88	2.22	✓
AD [56]	0.787	0.630	34.31	18.53	**0.21**	×
**Ours (DGADiff)**	**0.829**	**0.751**	**26.81**	**16.12**	0.26 ^†^	✓

↑: higher is better; ↓: lower is better. ✓ denotes training-free methods and × denotes methods that require training. The gray row highlights our method. ^†^ Diffusion-only time on RTX 4090. Full pipeline including SAM (VAE encode/decode + SAM mask generation + diffusion) is ∼2.3 s on RTX 5090. DGA ablation (removing DGA, a ZePo-like [35] controlled configuration under our unified backbone) is reported in Table 3a.

**Table 2:** Quantitative comparison on the large-scale benchmark (**1000 generated images**). For distribution metrics (ArtFID, FID), we report the **mean ± standard deviation** over 3 independent runs to ensure statistical stability.

Methods	ID Preservation	Style Consistency	Perceptual Distribution		Inference Time (s) ↓	Training-Free?
	FaceSim ↑	CLIP-I ↑	ArtFID ↓	FID ↓		
JoJoGAN [33]	0.801	0.642	30.55 ± 1.12	18.94 ± 0.85	0.50	×
Z-Star [18]	0.735	0.678	28.30 ± 0.75	17.85 ± 0.62	3.85	✓
StyID [19]	0.788	0.712	26.85 ± 0.82	18.22 ± 0.58	2.22	✓
AD [56]	0.782	0.628	32.74 ± 1.35	17.45 ± 0.80	**0.21**	×
**Ours (DGADiff)**	**0.822**	**0.748**	**24.95 ± 0.35**	**15.24 ± 0.28**	0.26 ^†^	✓

↑: higher is better; ↓: lower is better. ✓ denotes training-free methods and × denotes methods that require training. The gray row highlights our method. **Bold** and underline indicate the best and second-best results, respectively. ^†^ Diffusion-only time on RTX 4090. Full pipeline including SAM (VAE encode/decode + SAM mask generation + diffusion) is ∼2.3 s on RTX 5090. DGA ablation (removing DGA, a ZePo-like [35] controlled configuration under our unified backbone) is reported in Table 3a.

**Table 3:** Comprehensive ablation studies of DGADiff. We evaluate core components, mask fusion strategies, injection layers, and hyperparameter λ. Default settings of our full model are marked in gray; **bold** values indicate the best score within each sub-block. ↑ denotes higher is better, and ↓ denotes lower is better.

Ablation Settings	FaceSim ↑	ArtFID ↓	FID ↓	Time (s)
**(a) Core Components**				
w/o DGA module (ZePo-like config. [35])	0.797	28.54	17.17	**0.20**
w/o AdaIN Alignment	0.821	27.78	19.67	0.25
**Full Model (Ours)**	**0.829**	**26.81**	**16.12**	0.26
**(b) Mask Fusion Strategies**				
Linear Weighting (α=0.5)	0.805	28.10	17.50	0.26
Concatenation + Linear	0.790	29.30	18.20	0.28
Spatial Gating (Equation (4))	**0.829**	**26.81**	**16.12**	0.26
**(c) Injection Layer Locations**				
Early (Down blocks)	**0.835**	29.85	19.10	**0.23**
Late (Up blocks)	0.810	27.50	16.50	0.24
All Layers	0.827	26.75	**16.08**	0.35
Mid + Late (Default)	0.829	**26.81**	16.12	0.26
**(d) Sensitivity of Weight** λ				
λ=0.5 (Weak)	**0.840**	29.50	18.90	0.26
λ=1.5 (Strong)	0.775	28.00	17.10	0.26
λ=1.0 (Default)	0.829	**26.81**	**16.12**	0.26

## Data Availability

The original contributions presented in this study are included in the article. The portrait images used for training and evaluation are obtained from publicly available datasets: CelebA-HQ [47] and AAHQ [46]. Further inquiries can be directed to the corresponding author(s).
